# Commentary: a broader perspective on the RePAIR consensus guidelines (Responsibilities of Publishers, Agencies, Institutions, and Researchers in protecting the integrity of the research record)

**DOI:** 10.1186/s41073-018-0056-0

**Published:** 2018-12-19

**Authors:** Zoë H. Hammatt

**Affiliations:** Z Consulting, LLC, 49 South Kalaheo Avenue, Kailua, HI 96734 USA

The topic of responsibilities of publishers, agencies, institutions, and researchers in protecting the integrity of the research record is relevant for each of these stakeholders in the research enterprise. The RePAIR Consensus Guidelines reflect conversations on this important topic among diverse stakeholders rather than a single constituency. As such, they provide a starting point for additional discussion around improving communication among those handling retractions.

To advance the field beyond the Singapore and Montreal Statements and other referenced guidelines such as those produced by the Committee on Publication Ethics (COPE), the RePAIR Guidelines could serve as a springboard for articulating points of tension and offering solutions.

If these guidelines seek to offer specific recommendations on procedural aspects of interaction between stakeholders, however, extension beyond existing procedural guidelines (e.g., COPE and CLUE, referenced in the article) would be necessary. Such extension would require thorough literature review and additional consultation to ensure feasibility and a clear focus.

Most of the RePAIR guidelines are fairly general. Unlike the Singapore and Montreal Statements that emerged from the World Conferences on Research Integrity and set forth general principles of research integrity that transcend geographic and disciplinary boundaries, the RePAIR Consensus Guidelines reflect a US-centric approach, raising challenges for implementation in diverse contexts. Many “responsibilities” delineated for stakeholders arise from the US regulatory approach to handling research misconduct. One example is suggesting that regulatory or funding agencies should “notify [the] public of findings of research misconduct according to applicable federal or agency policy.” (p. 3). Since not all countries’ regulators or funding agencies publicize findings of research misconduct, further discussions could include exploration of whether making research misconduct findings public actually contributes to fostering a responsible research culture. If the guidelines were intended for application in a US setting, additional consultation with those handling alleged breaches of research integrity, along with legal counsel, editors and publishers, and researchers from various disciplines could lead to more precise guidance.

Another suggestion set forth in the RePAIR Guidelines is the notion that “Research Integrity Officers [should] ... ensure accurate reporting of data in submitted manuscripts.” (p. 4). While it is unrealistic to assume that a single individual can ensure accuracy in all manuscripts submitted by each member of a large research institution, the concept of institutional responsibility for accuracy of submissions is a critical one. Discussion of improvements to systems involving coordination between authors and relevant institutional offices could no doubt contribute to ensuring accuracy in manuscript submissions.

The RePAIR Guidelines would be significantly strengthened if practical recommendations could be articulated in connection with vague concepts, such as “protect whistleblowers.” These recommendations would need to address gaps in current guidelines and rest on a solid foundation of demonstrated effectiveness and comparative review. They would need to be described in sufficient detail so as to be practical and useful, and, where possible, relevant to diverse jurisdictions.

The suggestion of “overcoming barriers to communication” is a laudable goal that could serve as a springboard for future discussion. The RePAIR Consensus Guidelines present a starting point for discussion of novel and feasible solutions. With refinement by a diverse national or international group, such discussion could potentially lead to recommendations that could apply in either a country-specific setting or in a broader global context.

